# Supplementary material for: Content-rich biological network constructed by mining PubMed abstracts
Source: BMC Bioinformatics. 2004 Oct 8;5:147. doi: 10.1186/1471-2105-5-147 (PMC528731; doi:10.1186/1471-2105-5-147)
Supplement: Additional File 2 — The original results of the above study (non-essential files are deleted to keep the file size under the limit set by BMC bioinformatics). [file 1471-2105-5-147-S2.bz2 › chilibotAdditionalFile2/dip10/424ID11470772E10354/html/CCL1_CCR8.html]

 


 **CCL1** and **CCR8** 
  
Found 39 abstracts in PubMed, retrieved 10.  
 

 What does Google say? 
 PDF only 
| .edu only 

---

**Interactive relationship** (e.g. stimulation, inhibition, etc)

**Neutral relationship**- Further analysis demonstrated that  **CCL1**  activates the MAPK pathway in  **CCR8**  transfected CHO cells.  Ref: 12645948 Eur J Immunol, 2003
- ... interactions of CCR4 with MDC CCL22 or TARC CCL17,  **CCR8**  with I 309  [ **CCL1** ]   **CCL1** , and CRTH2 with prostaglandin D 2 play a critical role in the allergen induced recruitment of Th2 cells in the target tissues of allergic inflammation.  Ref: 12009564 Mol Immunol, 2002
- Th2 cells preferentially express  **CCR8**  and migrate selectively to its ligand, CC chemokine ligand CCL 1  [ **CCL1** ] .  Ref: 12707363 J Immunol, 2003
- The role of CCRS was supported by the observation that vMIP I, another  **CCR8**  ligand identified from the genome of a T cell transforming herpes virus, shared  **CCL1**  anti apoptotic activity.  Ref: 12645948 Eur J Immunol, 2003
- Increased responsiveness of murine eosinophils to MIP 1beta CCL4 and TCA 3  [ **CCL1** ]   **CCL1**  is mediated by their specific receptors, CCR5 and  **CCR8** .  Ref: 12050188 J Leukoc Biol, 2002

**Non-interactive relationship** (e.g. studied together, co-existance, homology, etc.)

- **CCR8** , the  **CCL1**  receptor, was identified on endothelial cells, and  **CCL1**  was found to be a chemoattractant for these cells.  Ref: 12489497 Isr Med Assoc J, 2002
- We show here that the  **CCR8**  restricted agonist Kaposi sarcoma associated herpesvirus encoded chemokine viral macrophage inflammatory protein 1 vMIP 1 rescues thymic lymphoma cells from dex induced apoptosis, similar to  **CCL1** ,.  Ref: 12525579 J Leukoc Biol, 2003
- The aim of our study was to characterize the role of the CC chemokine receptor 8  [ **CCR8** ]   **CCR8** , the only described receptor for  **CCL1** , in the rescue of murine thymic lymphoma cells and murine thymocytes from dexamethasone dex induced apoptosis.  Ref: 12525579 J Leukoc Biol, 2003
- Additionally, increased expression of the  **CCR8**  ligand, TCA 3  [ **CCL1** ] , has been detected in affected lungs in a mouse model of asthma.  Ref: 12496446 J Immunol, 2003
- Such pattern of  **CCL1**  responsiveness suggests that the  **CCR8**   **CCL1**  axis may regulate trafficking of cutaneous Treg and memory T cells into the skin.  Ref: 12442333 Eur J Immunol, 2002
- We studied the role of the  **CCR8**  ligand,  **CCL1** , in the specific recruitment of Th2 cells and eosinophils to the lung in a murine model of allergic airway disease.  Ref: 12707363 J Immunol, 2003
- Although it has been hypothesized that the rescuing effect of  **CCL1**  from apoptosis could be  **CCR8**  mediated, here, we formally demonstrate the role of such receptor as its selective antagonist encoded by the MC148 gene of molluscum contagiosum virus MC148 vMCC I inhibits v MIP 1 and  **CCL1**  induced rescue activity.  Ref: 12525579 J Leukoc Biol, 2003
- Using  **CCR8**  mice, evidence is provided that TCA3  [ **CCL1** ]   **CCR8**  interactions contribute to rapid onset CNS inflammation.  Ref: 12471141 J Immunol, 2002
- The  **CCR8**  ligand, TCA3  [ **CCL1** ] , is shown to be produced predominantly by resident microglia of the CNS in response to TNF.  Ref: 12471141 J Immunol, 2002
- Using antibodies specific for CCR5 and  **CCR8** , the chemotactic response to MIP 1beta and TCA 3  [ **CCL1** ] , respectively, was reduced significantly.  Ref: 12050188 J Leukoc Biol, 2002
- Here, we show that human peripheral blood CD4 and CD8 cutaneous CLA, but not intestinal memory integrin beta 7 nor IL 4 producing T cells, represent major subpopulations of circulating T cells that specifically migrate in response to the chemokine I 309  [ **CCL1** ]   **CCL1**  by virtue of  **CCR8**  expression.  Ref: 12442333 Eur J Immunol, 2002
